# An Estimation Algorithm for General Linear Single Particle Tracking Models with Time-Varying Parameters

**DOI:** 10.3390/molecules26040886

**Published:** 2021-02-08

**Authors:** Boris I. Godoy, Nicholas A. Vickers, Sean B. Andersson

**Affiliations:** 1 Department of Mechanical Engineering, Boston University, Boston, MA 02215, USA; bgodoy@bu.edu (B.I.G.); nvickers@bu.edu (N.A.V.); 2 Division of Systems Engineering, Boston University, Boston, MA 02215, USA

**Keywords:** single particle tracking, single molecule biophysics, fluorescence

## Abstract

Single Particle Tracking (SPT) is a powerful class of methods for studying the dynamics of biomolecules inside living cells. The techniques reveal the trajectories of individual particles, with a resolution well below the diffraction limit of light, and from them the parameters defining the motion model, such as diffusion coefficients and confinement lengths. Most existing algorithms assume these parameters are constant throughout an experiment. However, it has been demonstrated that they often vary with time as the tracked particles move through different regions in the cell or as conditions inside the cell change in response to stimuli. In this work, we propose an estimation algorithm to determine time-varying parameters of systems that discretely switch between different linear models of motion with Gaussian noise statistics, covering dynamics such as diffusion, directed motion, and Ornstein–Uhlenbeck dynamics. Our algorithm consists of three stages. In the first stage, we use a sliding window approach, combined with Expectation Maximization (EM) to determine maximum likelihood estimates of the parameters as a function of time. These results are only used to roughly estimate the number of model switches that occur in the data to guide the selection of algorithm parameters in the second stage. In the second stage, we use Change Detection (CD) techniques to identify where the models switch, taking advantage of the off-line nature of the analysis of SPT data to create non-causal algorithms with better precision than a purely causal approach. Finally, we apply EM to each set of data between the change points to determine final parameter estimates. We demonstrate our approach using experimental data generated in the lab under controlled conditions.

## 1. Introduction

Single Particle Tracking (SPT) is a class of experimental techniques and mathematical algorithms for following sub diffraction-limit sized particles moving inside living cells, including viruses, proteins, and strands of RNA [1,2,3]. Particles of this size cannot be resolved with standard optical microscopy, irrespective of the magnification. However, by labeling the particle (or particles) of interest with a fluorescent reporter such as a fluorescent protein or quantum dot, the motion of the tag and, by extension, the motion of the particle can be observed. While there are many different schemes, the general paradigm in SPT involves capturing a series of wide-field fluorescence images, localizing the fluorescent particle in each frame to form a trajectory, and then analyzing the trajectory to estimate motion model parameters.

There are a variety of motion models relevant to the biophysical application domain, including free diffusion, confined diffusion, directed motion, and combinations of these, such as joint diffusion and directed motion [4,5]. Given noisy observations of such a model (such as from a trajectory estimated by localizing a fluorescent particle in each frame of an image sequence), the most common technique to estimate the model parameters is to fit the chosen model to the Mean Square Displacement (MSD) curve. This very simple and popular approach has been enormously successful in probing biomolecular dynamics [6,7]. However, the resulting estimates depend on user choices such as the number of points to include when fitting the MSD data to the model, and the scheme does not account for many factors, including observation noise, motion blur arising from camera integration times, and other experimental realities [8]. While approaches based on optimal estimation, and in particular Maximum Likelihood (ML) techniques, are more complicated and often less intuitive than the MSD, they have a sound theoretical footing that guarantees efficiency and consistency of the estimates, and they consistently have been shown to outperform the MSD. ML algorithms have been developed for free diffusion [8,9,10,11], Ornstein–Uhlenbeck flow [12], and extended by one of the authors to more general motion models [13,14].

Most approaches, both those that use the MSD and those based on optimal estimation, typically assume that the model parameters, while unknown, are fixed. There have been some efforts on extending the analysis to determine the most likely model among a given set but these also assumed fixed parameters in each model with only one model active on the entire data set [5,15]. Other works have considered models that discretely switch between different motion models, each of which has a fixed (and unknown) set of parameters. One of the authors considered time-varying parameters using a jump Markov model [16] but such models impose a probabilistic structure on the changing parameter values that may be non-physical. In addition, they require a priori knowledge of the number of states. Other works have taken a data-driven technique to infer the number of states from the data directly [17,18]. However, these approaches have several parameters that need tuning and, while they are somewhat insensitive to that tuning, they also rely on a good choice of prior distributions on the parameter estimates. In [15], the problems of determining when a model switch occurred was separated from classifying what motion model is active but was not combined with optimal parameter estimation. Sliding (or rolling) windows have also been applied to SPT data, though with the single goal of segmenting the data based on features in the MSD [19,20,21,22]. More recently, machine learning techniques have been brought to bear on the problem of trajectories with time-varying parameters [23,24]. While results have been promising, there is a need to train the underlying neural networks and as a result there are concerns about transfer learning when applying the methods to different model classes than those used for training.

As with these prior works, the present paper focuses on systems that discretely jump between different parameter values and builds on our prior efforts using sliding windows and optimal estimation to produce an ML estimate of the parameters [25,26]. Here we describe a novel three stage algorithm that combines change detection to determine when model switches occur with ML estimation to find parameter estimates for each model. We assume each mode of motion is described by a general linear stochastic model with Gaussian noise statistics that encompasses a wide variety of dynamics. The first stage of our approach applies a sliding window to estimate the parameters of a general linear dynamic model at each point in time. These results are intended to help the user visualize the data and to inform the selection of tuning parameters for the second stage. In that second stage, we apply a Change Detection (CD) scheme to segment the data into regions with constant parameters. In the final step, we again determine ML estimates of the model parameters.

In both the first and third stages, the goal is to find an estimate of the (fixed) parameters defining a model over some given period of time, either a sliding window (stage one) or between time points where model changes were detected (stage three). Estimation of fixed model parameters from a set of measurements can be done using a variety of estimation methods, including fitting the desired model to the MSD, spectral factorization based on an auto-regressive, moving-average reformulation of Equation (1) [27,28], direct likelihood maximization using quasi-Newton numerical schemes [29,30,31], or Expectation Maximization (EM) [32]. Of these, MSD, despite its popularity, typically has the worst performance [10,13,14]. The others are all optimization based schemes and similar performance can be expected. EM, however, has at least two large benefits. First, in addition to producing (approximate) ML estimates of the parameters, it also yields the smoothed distributions on the particle locations from the data. Second, the approach is easily extended to nonlinear observation and motion models [13,14]. We therefore focus on this method as the core algorithm.

The second stage of our approach relies on CD for segmentation of the data. CD is a mature area and has been applied to a wide range of applications, including speech processing [33,34], image processing [35], analysis of electroencephalogram (EEG) and electrocardiogram (ECG) signals [36,37,38], and geophysics [35]. They have also been used in the context of SPT to segment trajectories based on model type, most commonly to distinguish between free and confined modes of motion [39,40,41]. CD methods use a threshold on a detection (residual) signal to indicate when a change has occurred and their performance depends heavily on the choice of that level. The main goal of the windowed estimation of stage 1 is to guide the user in the choice of the threshold. In this work we take advantage of the off-line nature of the analysis to use non-causal CD, averaging the results from forward and backward passes through the data to minimize the expected delay between the true and estimated change points.

Throughout the paper we use simulations to demonstrate and explore the elements of our three-stage method. We then validate the scheme using experimental data generated under controlled conditions that provide both real data and ground truth values through the use of a synthetic motion approach we have developed [42].

## 2. Methods

Data in SPT typically comes in the form of a sequence of camera images following both two-dimensional and three-dimensional motion. There are many algorithms for doing localization and linking [43,44] and we assume these steps have been performed prior to applying our approach. Note that while the focus of this paper is model parameter estimation, our algorithm does refine the given trajectories through the filtering and smoothing elements that are integral to our approach; details can be found in, e.g., [26]. The motion in each axis is assumed to be independent and described by a general linear time-varying model in each direction given by

(1) $$\begin{array}{cc} x_{k+1} & =a_{t}x_{k}+b_{t}+w_{k},\;w_{k}∼N\left(0,q_{k}\right),\\ y_{k} & =x_{k}+v_{k},\;v_{k}∼N\left(0,r_{k}\right), \end{array}$$

where *k* is the discrete time index, $x_{k}$, $y_{k}$, $w_{k}$, and $v_{k}$ are scalars, $q_{k}=2D_{k}\Delta t$ is the variance of the process noise defined by the diffusion coefficient $D_{k}$ and the sampling time Δt, and $r_{k}$ is the variance of the measurement noise as generated by a variety of processes, including shot noise due to the physics of photon generation in fluorescence and read-out noise in the camera. We note that there are important modes of motion in the biophysical domain that are not captured by Equation (1), and in particular those that are non-Gaussian in nature that need nonlinear or even non-Markovian models (see, e.g., [45,46,47]). However, system (1) can represent a variety of very relevant models in the SPT application. For example, setting $a_{k}\equiv 1,b_{k}\equiv 0$ describes pure diffusion; choosing $a_{k}<1$, $b_{k}\equiv 0$, yields an Ornstein–Uhlenbeck model that can capture tethered motion of a biomolecule or be used to approximate confined diffusion [12,48]. We further assume that the parameters are fixed for a finite interval of time before switching to different values for another interval. The number of switches is not known a priori.

**Figure 1:**
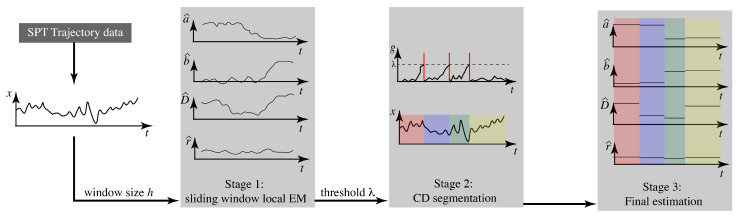
Overview of the three-stage algorithm for estimating parameters of a linear motion model that switches values at discrete times.

Our goal, then, is to determine the number of switches and to estimate the parameter values inside each interval. As described in Section 1, we take a three stage approach: (1) estimation of continuously varying parameters using sliding windows, (2) change detection for segmentation, and (3) parameter estimation on each interval. Our approach is illustrated in Figure 1. In what follows, we first describe the core elements of the three stages before bringing them together in the final algorithm.

### 2.1. Background on EM

As noted in Section 1, the EM algorithm is at the core of our technique. Here we give a brief overview of this well-known approach to ML estimation.

#### 2.1.1. EM for Fixed Parameter Estimation

Both the first and the last stage rely on using a set of *N* measurements, $Y_{N}=\left\{y_{1},y_{2},\cdots ,y_{N}\right\}$, to infer the parameters of the model in Equation (1). In the first stage, estimation inside each window assumes constant parameters, and in the last stage, estimation in each interval also assumes fixed parameters. In this section, then, we assume a time invariant model and note this by dropping the subscript *t* on the parameters.

The EM algorithm was introduced as a method for finding ML estimates when the likelihood function either could not be expressed in an analytical form or was too complex for direct optimization [32]. It is an iterative scheme that moves towards a local optimal of the likelihood. The essential idea is to use a so-called hidden variable, which in our case is taken to be the underlying particle trajectory $X_{N}=\left\{x_{1},x_{2},\cdots ,x_{N}\right\}$, to create an auxiliary function Q, which approximates the log-likelihood function. This function is defined as the conditional expectation of the joint log-likelihood of the observations and underlying trajectory,

(2) $$\begin{array}{c} Q\left(\theta ,{\hat{\theta }}^{\left(i\right)}\right)=E\left\{\log\left[p_{\theta }\left(X_{N},Y_{N}\right)\right]|Y_{N},{\hat{\theta }}^{\left(i\right)}\right\}, \end{array}$$

where ${\hat{\theta }}^{\left(i\right)}$ is the current estimate of the parameter. Calculating Q is referred to as the ‘E-step’ and depends on the conditional expectation given the complete data $Y_{N}$. For our model in Equation (1), the necessary distributions to calculate this expectation can be calculated using a Kalman filter and Kalman smoother. The next estimate of the parameters is then found through the ‘M-step’ by maximizing the auxiliary function

(3) $$\begin{array}{c} {\hat{\theta }}^{\left(i+1\right)}=\arg\;\max_{\theta }\;Q\left(\theta ,{\hat{\theta }}^{\left(i\right)}\right). \end{array}$$

For the general linear model considered here, the auxiliary function takes the form

(4) $$\begin{array}{c} Q(\theta ,{\hat{\theta }}^{\left(i\right)})=-N\log q^{-1}-N\log r^{-1}+\sum_{k=1}^{N}E\{{\left(y_{k}-x_{k}\right)}^{2}r^{-1}+{\left(x_{k+1}-ax_{k}-b\right)}^{2}q^{-1}|Y_{N},{\hat{\theta }}^{\left(i\right)}\}, \end{array}$$

where $\theta ={\left[\begin{array}{cccc} a & b & q^{-1} & r^{-1} \end{array}\right]}^{T}$. For further details and a robust numerical implementation of the EM algorithm, see [49].

#### 2.1.2. EM Using Local Likelihood

The essential idea behind the local likelihood approach is to do estimation inside a sliding window. As the window is slid along the data, an estimate of the parameter θ is produced at each point in time. It is important, however, to define these windows appropriately. The general local likelihood is given by

(5) $$\begin{array}{c} l_{t}\left(\theta_{t}\right)=\sum_{k=1}^{N}K_{k,t}l\left(y_{k}|\theta_{t}\right), \end{array}$$

where *h* is the window size, $l(y_{k}|\theta_{t})$ is the standard likelihood function, and

$$\begin{array}{c} K_{k,t}=K\left(\frac{k-t}{h}\right), \end{array}$$

is a kernel (also known as a weighting function) satisfying K(v)≥0 and $\int_{-\infty }^{{}_{\infty }}K\left(v\right)dv=1$. When selecting a kernel, it is important to use smooth windows to minimize an effect known as Gibbs ringing which causes oscillations in the time-varying estimate [50]. This can be achieved using kernels with rounded edges that progressively downweight data points far from the window center. A family of kernels defined by a parameter γ to achieve this is given by

(6) $$\begin{array}{c} K\left(v\right)=\left\{\begin{array}{cc} \frac{{\left(1-v^{2}\right)}^{\gamma }}{2^{2\gamma +1}B\left(\gamma +1,\gamma +1\right)}, & \mathrm{if}\;|v|\leq 1,\\ 0, & \mathrm{otherwise}, \end{array}\right. \end{array}$$

where $B\left(a,b\right)=\frac{\Gamma (a)\Gamma (b)}{\Gamma (a+b)}$ and Γ(·) is the standard gamma function. For γ={0,1,2}, we obtain what are known as the uniform, Epanechnikov, and biweight kernels, respectively.

Applying EM to this scenario simply means using the local likelihood to generate the auxiliary function. To distinguish it from the standard function in Equation (2), we denote it as $Q_{t}$. For the general linear system in Equation (1), we have

(7) $$\begin{array}{c} \begin{array}{cc} Q_{t}\left(\theta_{t},{\hat{\theta }}_{t}^{\left(i\right)}\right) & =-\left(\log q_{t}^{-1}+\log r_{t}^{-1}\right)+\sum_{k=1}^{N}K_{k,t}+q_{t}^{-1}\left[{\tilde{S}}_{11}-{\tilde{S}}_{01}^{T}\Gamma_{t}^{T}-\Gamma_{t}{\tilde{S}}_{01}+\Gamma_{t}{\tilde{S}}_{00}\Gamma_{t}^{T}\right]\\ \; & +r_{t}^{-1}\sum_{k=1}^{N}\left(K_{k,t}\left[{\left(y_{k}-{\hat{x}}_{k|h}\right)}^{2}+P_{k|h}\right]\right), \end{array} \end{array}$$

with

(8) $$\begin{array}{cc} {\tilde{S}}_{00} & =\sum_{k=1}^{N}\left[\begin{array}{cc} K_{k,t}\left[{\hat{x}}_{k|h}^{2}+P_{k|h}\right] & K_{k,t}{\hat{x}}_{k|h}\\ K_{k,t}{\hat{x}}_{k|h} & K_{k,t} \end{array}\right], \end{array}$$

(9) $$\begin{array}{cc} {\tilde{S}}_{11} & =\sum_{k=1}^{N}K_{k,t}\left({\hat{x}}_{k+1|h}^{2}+P_{k+1|h}\right), \end{array}$$

(10) $$\begin{array}{cc} {\tilde{S}}_{01} & =\sum_{k=1}^{N}\left[\begin{array}{c} K_{k,t}{\left[{\hat{x}}_{k+1|h}{\hat{x}}_{k|h}+P_{k+1,k|h}\right]}^{T}\\ K_{k,t}{\hat{x}}_{k+1|h} \end{array}\right]. \end{array}$$

Note that to arrive at the expressions above, we included the kernel function in Equations (7)–(10) and explicitly calculated the expected values in the auxiliary function. The conditional mean ${\hat{x}}_{k|h}$, covariance $P_{k|h}$, and cross covariance $P_{k,k-1|h}$, define the smoothed distribution on the underlying state and can be calculated from the Kalman filter and smoother (see, e.g., [51]). The inclusion of the parameter *h* in these expressions denotes that they depend upon the choice of window size. The maximization step can be performed explicitly by taking the derivatives of $Q_{t}$ with respect to the parameters, setting them to zero, and solving. Defining the vector $\Xi^{\left(i\right)}={\left[\begin{array}{cc} {\hat{a}}^{\left(i\right)} & {\hat{b}}^{\left(i\right)} \end{array}\right]}^{T}$, the resulting estimates are given by

(11) $$\begin{array}{cc} \Xi^{\left(i+1\right)} & ={\tilde{S}}_{01}{\tilde{S}}_{00}^{-1}, \end{array}$$

(12) $$\begin{array}{cc} {\hat{q}}_{t}^{\left(i+1\right)} & =\frac{1}{n}\left({\tilde{S}}_{11}-{\tilde{S}}_{01}^{T}{\left(\Xi_{t}^{\left(i+1\right)}\right)}^{T}-\Xi_{t}^{\left(i+1\right)}{\tilde{S}}_{01}-\Xi_{t}^{\left(i+1\right)}{\tilde{S}}_{00}{\left(\Xi_{t}^{\left(i+1\right)}\right)}^{T}\right), \end{array}$$

(13) $$\begin{array}{cc} {\hat{r}}_{t}^{\left(i+1\right)} & =\frac{1}{n}\sum_{k=1}^{N}K_{k,t}\left[\left(y_{k}-{\hat{x}}_{k|h}\right){\left(y_{k}-{\hat{x}}_{k|h}\right)}^{T}+P_{k|h}^{T}\right], \end{array}$$

where $n=\sum_{k=1}^{N}K_{k,t}$. Throughout this work we apply the Epanechnikov window (γ=1). Note that when working with a single, independent window (as in Phase 3 of our approach), it is reasonable to select a rectangular (uniform) window (γ=0). However, we found that because the change detection of stage 2 (described below) is not exact, better performance in terms of parameter estimation is achieved by downweighting samples at the edges as they may actually belong to a different model. Note that we have previously established that under this EM scheme, the local likelihood increases at each step and thus this local version inherits the results from standard EM that the algorithm will converge to at least a local maximum [26].

### 2.2. Algorithm Stages

#### 2.2.1. Stage 1: Sliding Window Estimation with Local Likelihood

The first stage of our approach is to apply EM with local likelihood using a sliding window approach. As noted above, the role of this stage is only to inform the user about likely changes in the dynamics to guide the selection of thresholds for use in automatic change detection (described in Section 2.3 below), not to produce any final estimates. To demonstrate this stage, we generated realizations of a system given by Equation (1) that switched the diffusion coefficient from D=0.1 to D=0.2 μm${}^{2}$/s, sampled at a rate of Δt=0.1 s and with the other parameters fixed at a=1, b=0, and r=0.1. For simplicity, we assumed the fixed constants *a*, *b*, and *r* were all known and only *D* needed to be estimated. We then processed the data using both a rectangular window (which we refer to as the naive approach) and an Epanechnikov window.

For this simple setting of only estimating the covariance of the process noise (that is, the diffusion coefficient), there are multiple adaptive filtering algorithms, dating back to the 60’s (see, e.g., [52,53]). A recent version of these methods that minimized a quadratic function of the innovations (that is, the difference between the predicted and actual measurements) was introduced in [54]. Based on the Kalman filter, this method was shown to outperform prior techniques. We therefore compare our results to the algorithm in [54] as a benchmark in this simple setting of diffusion-only estimation. It is important to note, however, that our algorithm is more general as it is able to estimate all the model parameters.

**Figure 2:**
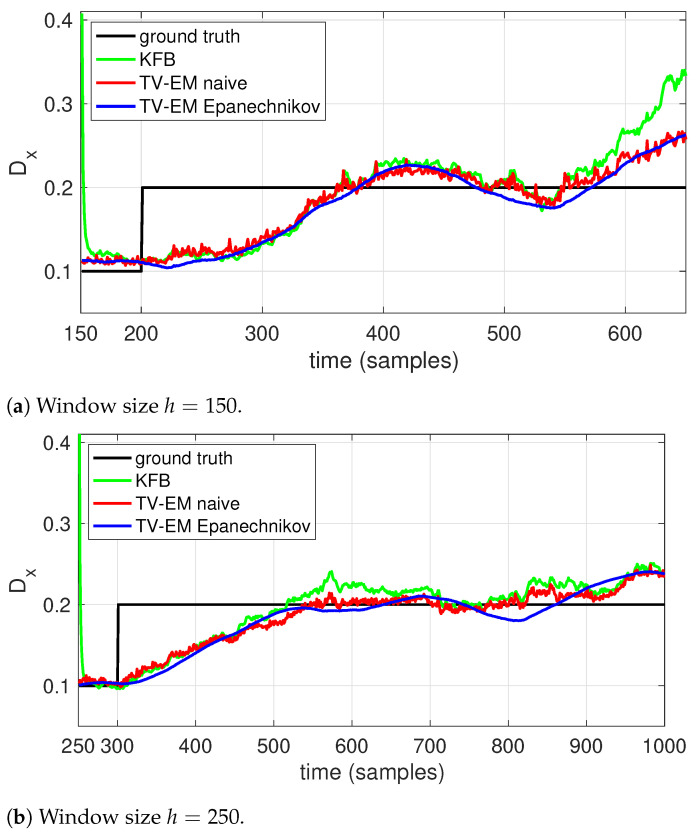
Estimating the diffusion coefficient (solid black) using local EM with (blue) an Epanechnikov window, (red) a naive, rectangular window, as well as using a (green) a Kalman filter based scheme. Estimation was done using a window size of (**a**) *h* = 150 and (**b**) *h* = 250.

The results are shown in Figure 2 for two different data sets, one with *D* changing at time 200 and one with the change at time 300. Each set consists of 10 realizations of the trajectories and the results shown in Figure 2 are averaged over those ten runs. To highlight the effect of the window size, we used h=150 on the first data set and h=250 on the second. As expected, with the shorter window all algorithms show a faster response. Both EM methods show a smoother response and better accuracy than the Kalman filter-based scheme. While both windows have similar behavior, the use of an Epanechnikov window clearly helps to smooth out the estimate relative to the naive, rectangular window, though the effect is more muted with the longer window.

#### 2.2.2. Stage 2: Change Detection

Given a sequence of random variables $y_{n}$ from a probability density function dependent on a parameter θ, CD aims to find the unknown time $t_{c}$ such that the (vector) parameter $\theta =\theta_{o}$ for $t<t_{c}$, and $\theta =\theta_{1}$(≠$\theta_{o}$) for $t>=t_{c}$. There is a rich literature on CD and many different techniques have been developed for a wide variety of settings (see, e.g., [55,56]). In general, the idea behind CD is to first define a *residual signal* that is close to zero when the parameter has not changed and that rapidly increases after a change, and then to define a decision rule which monitors the residual and declares when a change has occurred. In the SPT setting, the problem is particularly challenging due to the need to detect non-additive spectral changes in the model since the diffusion coefficient enters through the variance of the input noise.

CD relies on a model describing how the parameter affects the measurements. While our SPT data is described by the system in Equation (1), CD is difficult to apply to such state-space formulations. For this second stage, then, we choose to describe our measurements with a simple Autoregressive with Exogenous Input (ARX) model as these have been shown to be effective for capturing spectral changes [37,57]. There are more general models of this type that have been applied to parameter identification in SPT, including for anomalous diffusion based on fractional dynamics in single particle trajectories [58,59]. However, the goal of this stage is just CD, not model identification, and the ARX model provides a simple approach with well-established theory for finding change points in the data.

ARX models of order *p* are given by

(14) $$\begin{array}{c} y_{k}=\sum_{i=1}^{p}a_{i}^{j}y_{k-i}+\epsilon_{k},\;\mathrm{var}\left\{\epsilon_{k}\right\}=\sigma_{\epsilon }^{2}, \end{array}$$

where *j* is an index indicating a specific model and $a^{j}=\left(a_{1}^{j},\;a_{2}^{j},\;\cdots ,\;a_{p}^{j}\right)$ are the model parameters for that model. Given a set of data, we can automatically determine the best order *p* for a model using the Bayesian Information Criterion (BIC) as follows. The BIC is defined by

(15) $$\begin{array}{c} BIC\left(p\right)=-2l\left(\hat{\theta }\right)+p\log N, \end{array}$$

where l(θ) is the log-likelihood, *N* is the number of data points, *p* the model order, and $\hat{\theta }$ the ML estimate of the parameter. The model order is then selected as the one minimizing the BIC.

**Figure 3:**
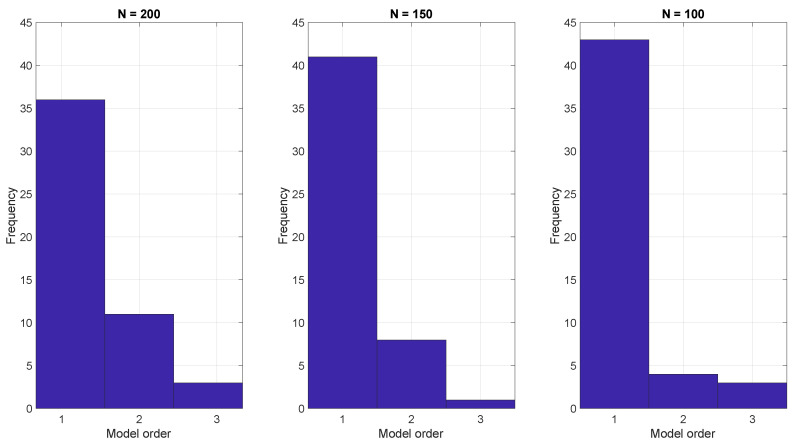
ARX model estimation based on the BIC criterion with run lengths of (**left**) 200, (**center**) 150, and (**right**) 100.

To demonstrate the ARX model approach, we generated 50 realizations using Equation (1) with three different run lengths and then estimated the parameters of the ARX model Equation (14) using the BIC. The resulting model orders that optimized the BIC are shown in Figure 3. In all cases the BIC indicates that a low order model, often with just one parameter, is sufficient.

For the residual signal we use a sufficient statistic for detecting spectral changes known as the cumulative sum (CUSUM) (see [56,60]). CUSUM can be defined in a few different ways; here we use a version based on the likelihood ratio given by the general formulation

(16) $$\begin{array}{cc} s_{k} & =\log\frac{p_{\theta_{1}}\left(y_{k}|Y_{k-1}\right)}{p_{\theta_{o}}\left(y_{k}|Y_{k-1},\right)},\;g_{k}={\left(g_{k-1}+s_{k}\right)}^{+}, \end{array}$$

where $Y_{k-1}$ is all the data up to time k−1, ${\left(\cdot \right)}^{+}=\max\left\{\cdot ,0\right\}$, and $\theta_{o}$ and $\theta_{1}$ are the parameter values before and after the change, respectively. Finally, we define the decision rule by selecting a threshold λ such that a parameter change is declared to occur at the time when $g_{k}$ exceeds that threshold,

(17) $$\begin{array}{c} {\hat{t}}_{c}=\min\left\{k:g_{k}\geq \lambda \right\}. \end{array}$$

As written in Equations (16) and (17), the CUSUM test assumes prior knowledge of the parameter values before and after the change. In our setting, of course, these values are not known ahead of time. We overcome this by simultaneously estimating two ARX models of the form in Equation (14). The first of these, termed $M_{\theta_{0}}$ is a long term model that is estimated using all of the data from the most recently detected change up to the current time. The second, $M_{\theta_{1}}$ is estimated using data from a sliding window of size *h*. Inserting the ARX models in Equation (16) yields the CUSUM signals for our setting,

(18) $$\begin{array}{c} s_{k}=\frac{1}{2}\log\frac{\sigma_{\epsilon_{0}}^{2}}{\sigma_{\epsilon_{1}}^{2}}+\frac{{\left(e_{k}^{0}\right)}^{2}}{2\sigma_{\epsilon_{0}}^{2}}-\frac{{\left(e_{k}^{1}\right)}^{2}}{2\sigma_{\epsilon_{1}}^{2}},\;g_{k}=\sum_{i=1}^{n}s_{k}, \end{array}$$

where $e_{k}^{i}$ is the residual error of the $i^{\mathrm{th}}$ model given by the difference between the predicted and true measurement at time *k*. The two models are compared using the CUSUM test with a threshold λ selected by the user. Once a parameter change is detected, both models are reset, the prior data is discarded, and the process started again to search for the next change point. The threshold λ is tuned by the user to achieve satisfactory results, guided by the expected number of model changes as indicated by Stage 1 of our approach.

In general, there is a delay between the actual change and the point of detection imposed by the time it takes the residual signal to grow. This is mitigated somewhat by the fact that the time assigned to the detected change corresponds to the beginning of the sliding window of the second model but in general still leads to a bias in the estimated change time. When detecting changes online, this bias can be reduced by increasing the sensitivity of CD by selecting a lower threshold at the cost of possibly increasing false positives in CD. In the SPT context, however, estimation is typically done offline in post-processing. We take advantage of this to reduce the bias in estimating the change time by performing two passes on the data, one forward in time and one backward in time. CD points from the two passes that are close enough (as defined by the user) are averaged to reduce the bias. If there is no clear match then the CD from the forward pass is selected.

**Figure 4:**
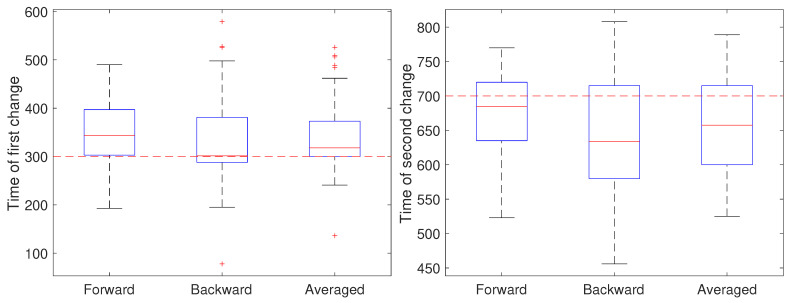
Detected time of change under a low noise setting based on using only the forward pass, only the backward pass, or averaging the two. (**left**) Estimates of the time of the first change (at k=300) of the parameter *a* from a value of 1 to 0.8 and (**right**) estimates of the time of the second change (at k=700) back to 1.

**Figure 5:**
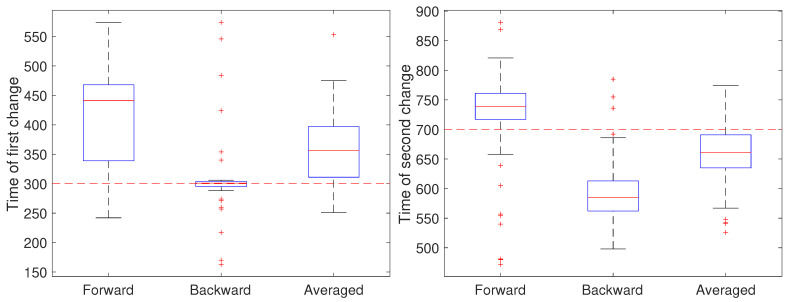
Detected time of change under a high noise setting based on using only the forward pass, only the backward pass, or averaging the two. (**left**) Estimates of the time of the first change (at k=300) of the parameter *a* from a value of 1 to 0.8 and (**right**) estimates of the time of the second change (at k=700) back to 1.

To illustrate the CD stage, we performed two sets of 50 independent simulations of the system in Equation (1) at a measurement noise level of r=0.001 μm${}^{2}$ (corresponding to a localization precision of approximately 32 nm), with a sampling rate of 0.1 s and a total trajectory of 100 s. In the first setting, all parameters except for *a* were held fixed at b=0 and D=0.05 μm${}^{2}$/s. Initially, the remaining parameter was set to a=1, switched to a=0.8 after 30 s (k=300) and returned to a=1 at 70 s (k=700). The results are shown in Figure 4. In these simulations, there is some improvement in the detection time of the first change when using the averaged result. For the second change, however, results based on just the forward pass were better than either the backward or averaged. As the measurement noise is increased, though, detection becomes more challenging and a larger delay is expected since the threshold will likely need to be set higher to avoid false positives. To explore this, we ran another set of 50 trials but with a measurement noise of r=0.01 μm${}^{2}$/s (corresponding to a localization precision of 100 nm). These results are shown in Figure 5. Under this setting, both the forward and backward passes have larger bias, larger variance, and many more outliers than in the low noise setting and the results based on the averaged value are more reliable.

**Figure 6:**
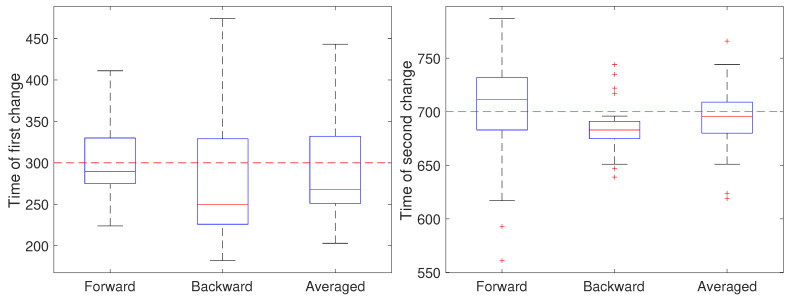
Detected time of change under a low noise setting based on using only the forward pass, only the backward pass, or averaging the two for the (**left**) first change (at k=300) of the diffusion coefficient from 0.05 to 0.2 μm${}^{2}$/s and the (**right**) second change (at k=700) back to 0.05 μm${}^{2}$/s.

**Figure 7:**
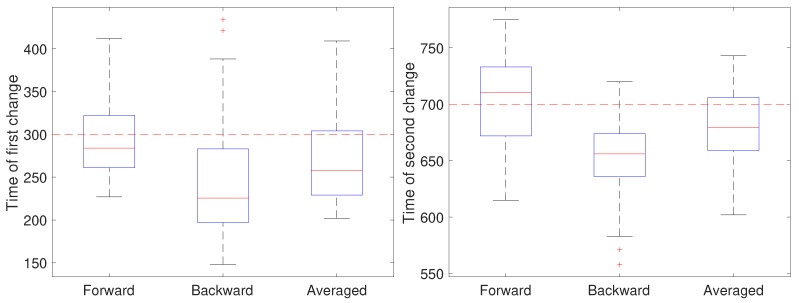
Detected time of change under a high noise setting based on using only the forward pass, only the backward pass, or averaging the two for the (**left**) first change (at k=300) of the diffusion coefficient from 0.05 to 0.2 μm${}^{2}$/s and the (**right**) second change (at k=700) back to 0.05 μm${}^{2}$/s.

In the second setting, all parameters except the diffusion coefficient were held fixed at a=1 and b=0 and the measurement noise was again set to r=0.001 μm${}^{2}$. The diffusion coefficient was initially set to 0.05 μm${}^{2}$/s, switched after 30 s (at k=300) to 0.2 μm${}^{2}$/s for the next 40 s, and switch back to 0.05 μm${}^{2}$/s (at k=700). The results are shown in Figure 6. Note that for the first change, the forward pass yields the most accurate estimate of the change while for the second change the backward pass is more accurate. We also ran a second set of 50 trials at the larger noise of r=0.01 μm${}^{2}$; these results are shown in Figure 7 and show a similar effect with respect to the forward, backward, or averaged results as in the low noise setting. This reflects the fact that in general detecting an *increase* in a covariance parameter is easier than detecting a decrease. Without prior knowledge of the change, the average of the forward and backward estimates provides a robust result. Of course, the windowed estimates of the first stage of our approach could be used to estimate the direction of change and the thus guide the user to choose either the forward or backward CD result.

#### 2.2.3. Stage 3: Final Estimation and the Complete Algorithm

The final stage is simply to run the EM algorithm on each set of data between the detected change points to produce the final estimates. The three stage algorithm, illustrated in Figure 1, thus proceeds as follows. Given a trajectory of single particle tracking data, the user first selects a window size *h* and runs local EM to produce continuous estimates of the parameters. Shorter windows are of course more sensitive to parameter changes but less robust to noise. While there are data-driven methods for selecting an appropriate window size such as the Steins Unbiased Risk Estimator (SURE) [61], the results of this stage are used only to roughly estimate the number of parameter changes and a trial-and-error approach driven by domain knowledge and experience will likely be sufficient. In Stage 2, the user selects a threshold λ for the CUSUM test. As with the window size, some trial-and-error is likely needed to determine a good threshold but this choice is now informed by the expected number of changes indicated by the Stage 1 result. After running CD, the original data can be segmented into windows of maximal length, each of which has a fixed model. In Stage 3, the constant parameter EM algorithm in Equations (2) and (3) is then run on each segment independently to determine the final estimates.

### 2.3. Generation of Synthetic Data

**Figure 8:**
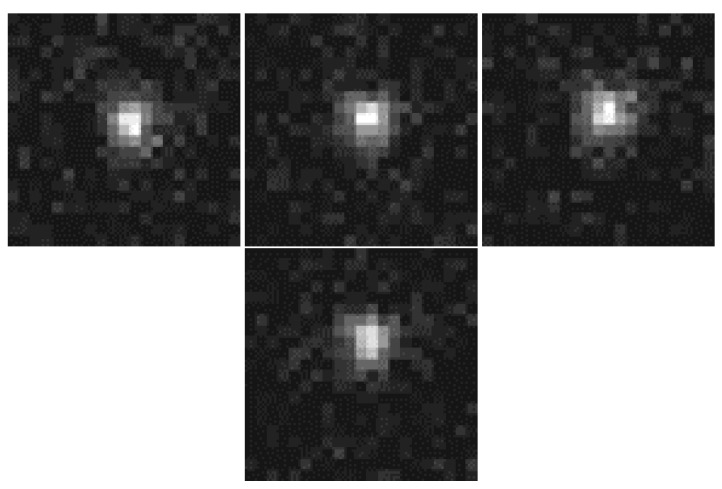
Four sequential segmented image frames from a synthetic motion sequence.

While simulations can be very useful to explore algorithm efficacy, it is important to test algorithms on realistic data, ideally with accompanying ground truth. Our experimental procedure to achieve this, known as synthetic motion and described in detail in [42], consists of four steps: (1) generate numerical sample paths using the motion model in Equation (1) for a given set of parameters, (2) control the motion of a fluorescent particle (such as a quantum dot or fluorescent microsphere affixed to a coverslip) using a piezoelectric stage with nanometer-scale precision, (3) acquire images of the moving particle using a widefield microscope, and (4) process the resulting images to generate a measured trajectory. Our specific implementation uses a high speed 3D piezostage (Nano-PDQ, Mad City Labs) mounted on an inverted optical microscope (Zeiss Axiovert 200) and controlled using a custom-designed controller to achieve both high speed and precision (below 10 nm) in positioning. The controller was implemented on an field programmable gate array (FPGA) on a National Instruments compact Reconfigurable Input Output system (NI cRIO 9076). Particle motion was observed using a 63×, 1.2 N.A. water immersion objective and diffraction-limited images captured using an sCMOS camera (Prime 95B, Photometrics). To avoid motion artifacts, the piezostage was moved and allowed to settle at the next position in the trajectory while the camera was offloading the previous image and held stationary during the next image acquisition. Acquired images were then segmented and the location of the fluorescent particle in each frame estimated using a nonlinear least-squares fit to a Gaussian profile [62]. Four sequential frames from a typical synthetic data set are shown in Figure 8.

## 3. Results and Discussion

We generated 90 trajectories of synthetic data with a step size of Δt=0.1 s, each consisting of 1000 frames. The parameters $a_{t}$, $b_{t}$, and $D_{t}$ were set as shown in Table 1, corresponding to pure diffusion for 250 steps, an Orenstein–Uhlenbeck (O-U) motion for the next 250 steps, fixed motion for 250 steps, and finally directed motion with diffusion for the final 250 steps. The observation noise $r_{t}$ was determined by the experimental conditions. The values during the first three phases were selected based on classification results for the motion of the transmembrane protein CD44 on the surface of macrophages as reported in [15] while the values for the final, directed motion stage correspond to a speed of 2 μm/s, consistent with the speed of dynein on microtubules and inspired by early results of SPT in virus tracking [63].

**Figure 9:**
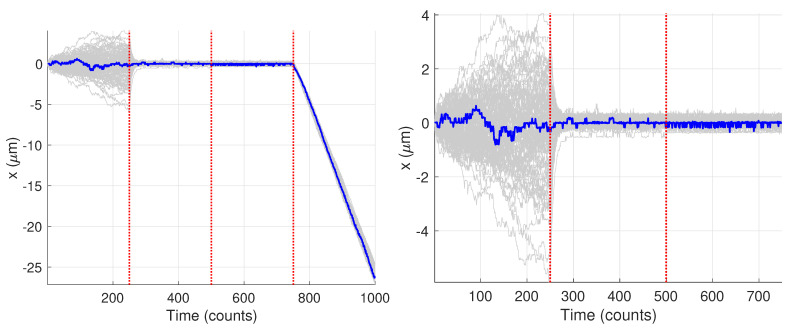
Synthetic motion trajectories generated from image data over (**left**) the full 1000 frames and (**right**) over the first 750 frames to highlight the diffusive nature of the motion. The example trajectory used is highlighed in blue. Vertical red dashed lines are where the model values changed according to the values in Table 1.

Figure 9 shows the generated trajectories and a zoom-in on those trajectories over the first 750 data points. Note that these trajectories are those produced from the Gaussian fitting-based analysis of the image data; see Section 2.3. The trajectories show a clear diffusive motion in the first 250 steps and then a transition to the tethered motion. Visually there is only a small difference between the OU motion and the fixed motion due to the small diffusion coefficient during the O-U phase and the measurement noise. In what follows we first carry out the analysis for the single specific trajectory shown in blue.

**Figure 10:**
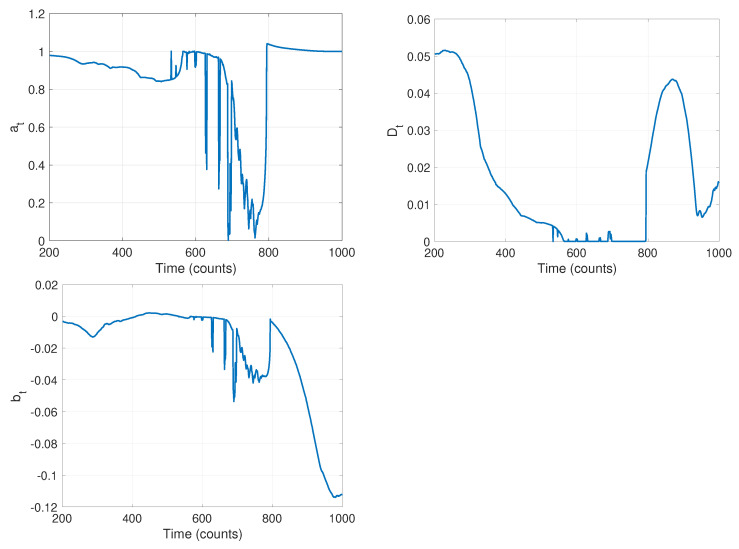
Results of stage 1: time varying estimates of parameters (**left**) *a*, (**center**) *D*, and (**right**) *b*, using a sliding window of size h=200.

The first stage of our algorithm is to run the local-likelihood based estimation algorithm. For this we chose a window of size h=200; the resulting time-varying estimates of the parameters are shown in Figure 10. Note that while we are estimating all four parameters of the model in Equation (1), we do not expect the measurement noise to change and thus look only at the parameters $a_{t}$, $D_{t}$, and $b_{t}$ in this stage. The curves in Figure 10 clearly indicate that there are likely model changes occurring, though they do not clearly reveal where or how many. From the $a_{t}$ curve, it seems reasonable to infer 2–3 changes with switches at approximately k=500, k=700, and k=800. The value of $D_{t}$ seems to go through two changes at the approximate times k=250 and k=800. Finally, $b_{t}$ seems to change values twice, once near k=700 and again near k=800. Combining these, and recognizing that multiple parameter values may change in a model switch, Stage 1 indicates there are likely three changes in this data set.

**Figure 11:**
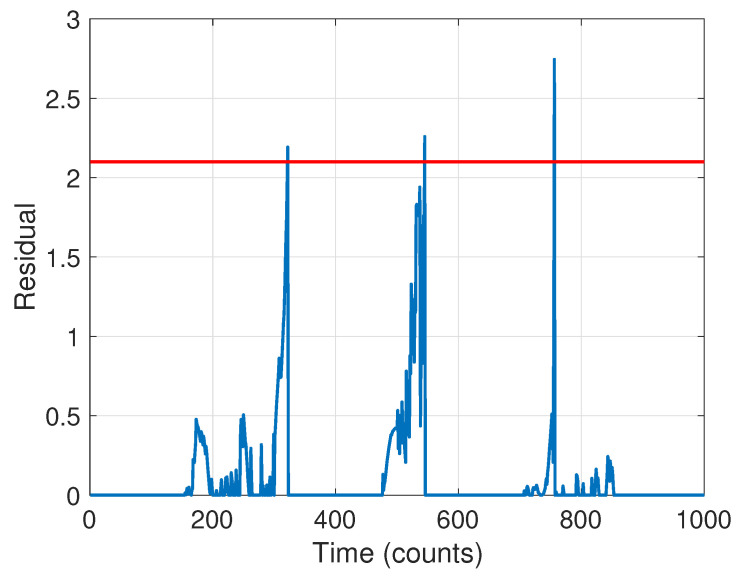
Residual signal for change detection. The selected threshold is indicated with a red dashed line.

The next stage is to run the CD scheme. The BIC criterion led to an ARX model order of p=1. The resulting residual signal in Equation (18) for the forward pass is shown in Figure 11 (the residual for the backward pass is qualitatively similar). Guided by the results of Stage 1, we selected a threshold of λ=2.1, leading to a detection of three changes. The change times based on the forward pass were found to be at time steps [321, 544, 755]; using the backward pass only they were at [247, 422, 735]. The final detection times were thus taken to be the average of these, [284, 483, 745], quite close to the true times of [250, 500, 750].

The final stage is then to run the EM-based ML estimation in each segment. The resulting parameter values are given in Table 2. For the parameters with ground truth, the percent error is also given in the table. In general, the results are quite good. The one exception is in the value of $a_{t}$ in the third stage where the particle is fixed. This is discussed a bit more below after analyzing the results for all the trajectories.

We then applied our algorithm to all 90 trajectories in the synthetic motion data set we created. Since in practice it is unlikely one would tune the algorithm for each trajectory when analyzing large data sets, we applied the same ARX model order (p=1) and threshold (λ = 2.1) to every trajectory (better results would be expected, of course, if each trajectory was handled independently). Using these settings, the CD identified three changes in 64 of the trajectories and two changes in the remaining 26.

**Figure 12:**
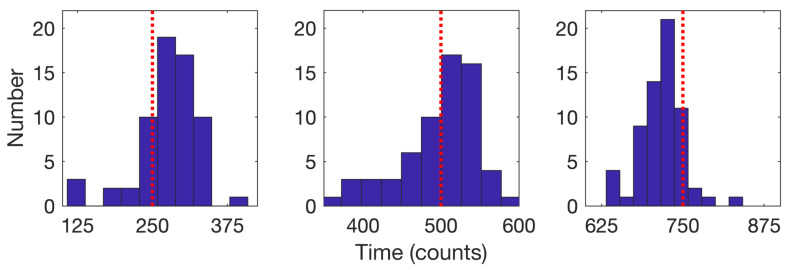
Histograms of the detected times of model changes when three changes were detected (64 trajectories) for the (**left**) first, (**center**) second, and (**right**) third change. Red dotted lines indicate the true change times of 250, 500, and 750. Mean detected times are 276.2 for the first, 497.1 for the second, and 717 for the third.

We consider first the 64 trajectories with three detected changes. Histograms for the estimated times of the model change based on the average of the forward and backward passes are shown in Figure 12 for the cases with three changes; the mean detected times were $k_{1}=276.2$, $k_{2}=497.1$, and $k_{3}=717$.

**Figure 13:**
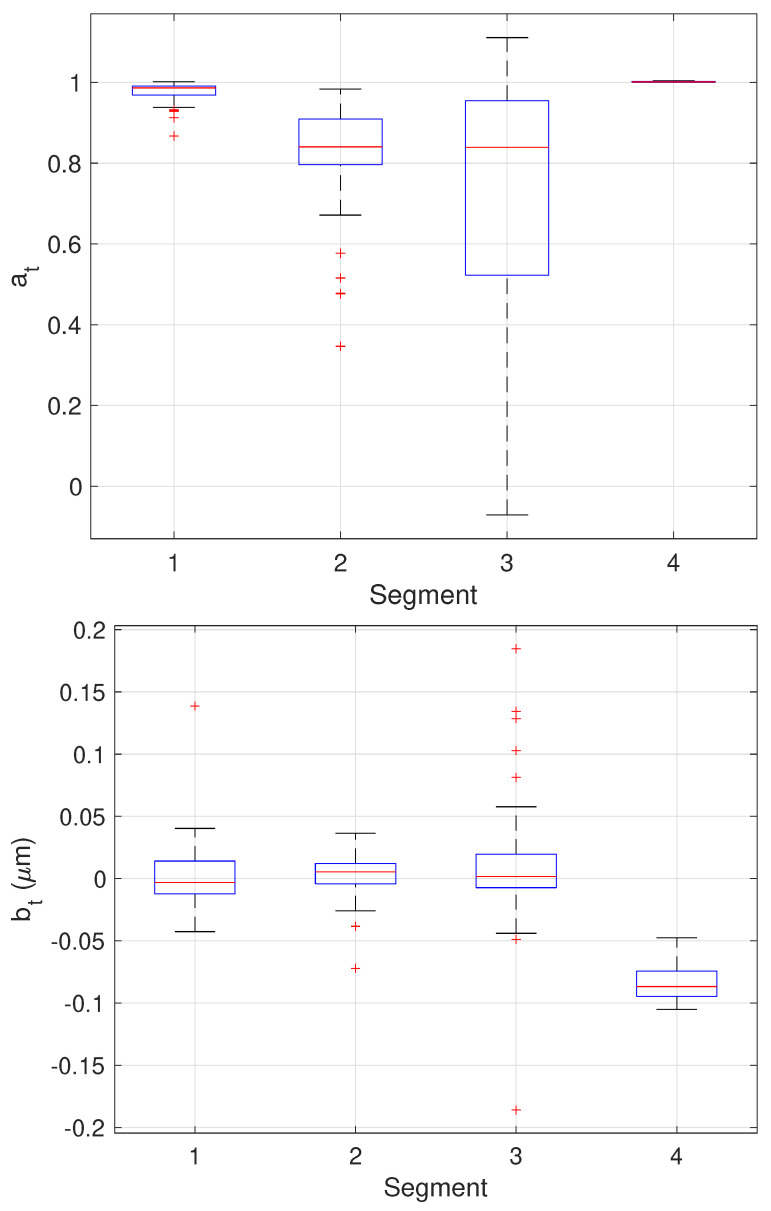

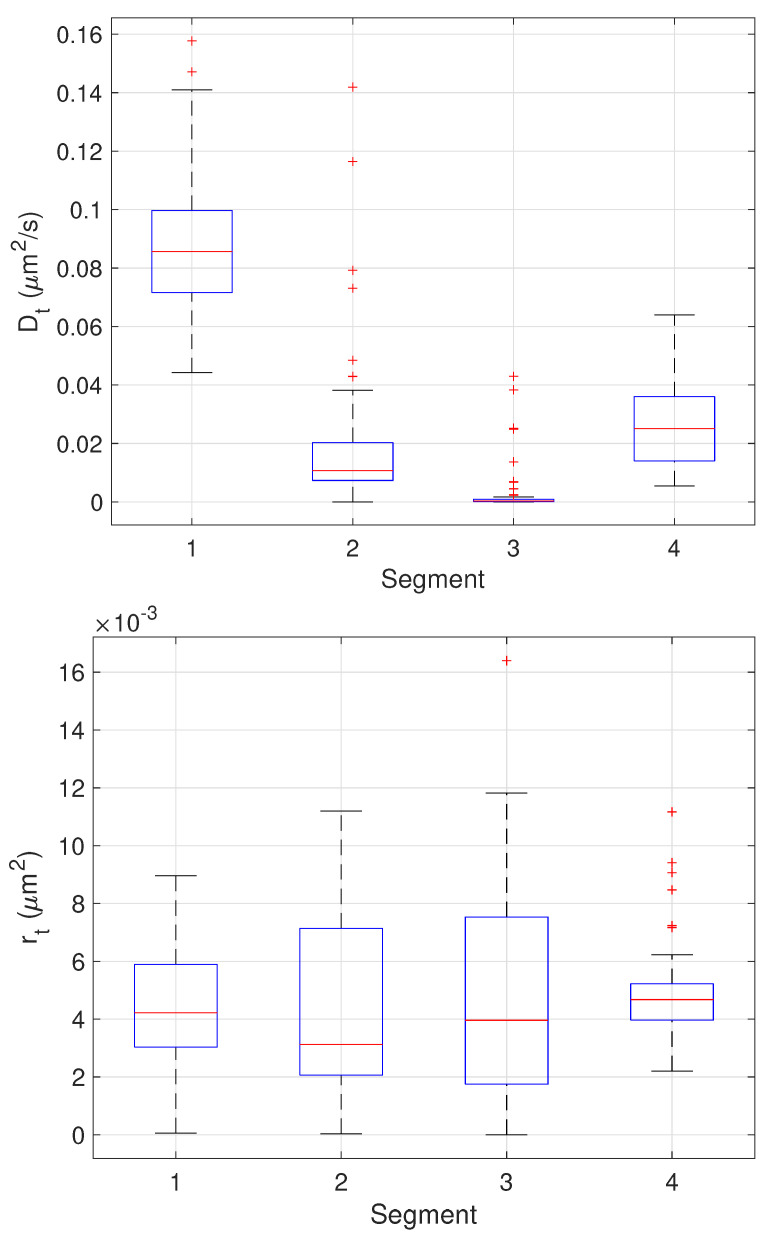
Box plots showing median, center two quartiles, and outliers for the estimates of the model parameters in each segment for the 64 trajectories considered.

The results of the final stage of our algorithm on the 64 trajectories yielding three change points are shown as boxplots in Figure 13 while the mean and standard deviation of the estimates are shown in Table 3. Since these trajectories yielded three changes, the estimated values can be compared directly to the ground truth values. These results show very good performance across all parameter estimates with the one exception being the values of $a_{t}$ in the third stage. In this stage, the particle is fixed. The EM algorithm, however, assumes some amount of stochasticity in the model (that is, that $D_{t}$ should not be exactly zero) and appears to compensate for this by biasing $a_{t}$ toward smaller values; this would correspond to a larger restoring force that keeps the particle near zero. While we do not have ground truth for the measurement noise $r_{t}$, the estimates correspond to a localization error of 60–70 nm which is reasonable for the imaging conditions in the synthetic motion data.

**Figure 14:**
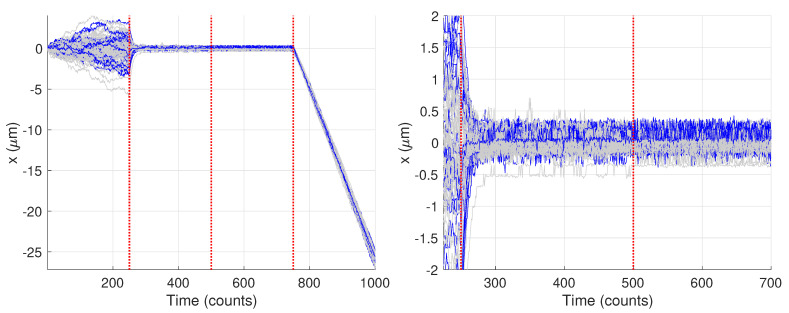
Synthetic motion trajectories generated from image data with the 26 trajectories yielding only two changes highlighted in blue. Vertical red dashed lines are where the model values changed. (**left**) Full trajectories. (**right**) Zoom in to highlight differences between the blue and gray trajectories.

The remaining 26 trajectories showed only two changes. Figure 14 highlights the trajectories where only two changes were found, with the remaining shown in light gray. Interestingly, this particular set of trajectories does show a marked difference from the other trajectories. In particular, there appears to be higher noise in the data which obscures the differences between the second set of model parameters (from times 250–500) and the third (from times 500–750). The blue curves look qualitatively similar throughout that entire time span while the trajectories in gray show a clear diminishing of motion after time 500. Because results from these curves could not be compared against the ground truth, they were not analyzed further.

## 4. Conclusions

We described and demonstrated a three-stage algorithm for analyzing single particle tracking data for systems with time-varying parameters that switch between (unknown) discrete values. Our approach depends on two primary parameters, a window size for the first stage and a detection threshold for the second. In general, the choice of window size depends on user experience and expected rate of change of model parameters. A smaller window size will be more sensitive to changes but may also produce false positives. However, since the results of this stage are only used to guide the selection of the threshold parameter, there is some amount of insensitivity to its choice. The second parameter is the threshold on the CUSUM signal for change detection and should be selected based on the number of changes expected in the data, informed by the windowed estimation of the first stage. In practice, users may wish to cycle between the first two stages of the algorithm before setting on a final window size. Finally, the data is segmented into regions of fixed parameters and EM applied one last time to determine the model estimates. We demonstrated the approach using data from a synthetic motion technique that provides both experimental measurements and ground truth values. These results produced accurate parameter estimates and also identified trajectories where the synthetic data did not quite match the expected model.

This work focused on a general linear model with Gaussian noise for describing the particle motion. There are at least three natural extensions. For the first, a fourth stage where the results of the third stage are used to guide selection of a specific model (e.g., selecting pure diffusion will set a=1 and b=0) to reduce the number of parameters that need to be estimated. Running EM estimation once again but now for the more limited model should yield a refined estimate of the model parameters. The second extension is to replace the linear dynamics and Gaussian statistics with a more general parameterized model that encompasses nonlinear motion, nonlinear observations, and non-Gaussian statistics. This could be used, for example, to estimate parameters of a confined diffusion model directly (rather than approximating such motion with an O-U model), to allow for different classes of anomalous dynamics, or to eliminate the need for applying an external localization algorithm as the EM algorithm will produce trajectories in addition to ML estimates of the model parameters; see [64] for initial work along these lines. The third extension is to handle missing data points in the particle trajectories. Our current approach assumes a constant time step in between each point on the trajectory. It is not uncommon in SPT data that some points are missing, either due to errors in localization, fluctuations in signal intensity, or other issues and extending our method to allow for known but non-constant time steps along the trajectory would expand the types of data that could be analyzed.

## Figures and Tables

**Table 1:** Synthetic motion parameter values.

	Time Step			
Parameter	1–250	251–500	501–750	751–1000
$a_{t}$ [unitless]	1	0.86	1	1
$b_{t}$ [μm]	0	0	0	−0.1
$D_{t}$ [μm${}^{2}$/s]	0.1	0.01	0	0.01

**Table 2:** Estimated motion parameter values and percent error from ground truth.

	Time Step			
Parameter	1–284	285–483	483–745	746–1000
$a_{t}$ [unitless]	0.9691 (3.1% error)	0.8661 (0.7% error)	0.2482 (75.2% error)	1.0006 (0.06% error)
True $a_{t}$	1.0	0.86	1.0	1.0
$b_{t}$ [μm]	−0.0015 (1.5% error)	0.0007 (0.7% error)	−0.0463 (4.63% error)	−0.098 (2.0% error)
True $b_{t}$	0	0	0	−0.1
$D_{t}$ [μm${}^{2}$/s]	0.0408 (52% error)	0.0064 (36% error)	0.0094 (N/A % error)	0.0112 (12% error)
True $D_{t}$	0.1	0.01	0	0.01
$r_{t}$ [μm${}^{2}$]	0.0041	0.002	0.0054	0.0052

**Table 3:** Mean and standard deviation of the motion parameter values in each segment over the 64 trajectories with 3 detected changes, together with the true values.

Parameter	Segment 1	Segment 2	Segment 3	Segment 4
$a_{t}$ [unitless]	0.9768 ± 0.0237	0.8282 ± 0.127	0.7085 ± 0.3185	1.002 ± 0.0011
True $a_{t}$	1.0	0.86	1.0	1.0
$b_{t}$ [μm]	0.0011 ± 0.025	0.0029 ± 0.0165	0.0109 ± 0.047	−0.0836 ± 0.015
True $b_{t}$	0	0	0	−0.1
$D_{t}$ [μm${}^{2}$/s]	0.0874 ± 0.023	0.01934 ± 0.025	0.0030 ± 0.0083	0.0264 ± 0.0141
True $D_{t}$	0.1	0.01	0	0.01
$r_{t}$ [μm${}^{2}$]	0.0045 ± 0.0022	0.0045 ± 0.0031	0.0046 ± 0.0034	0.0049 ± 0.0016

## Data Availability

No new data were created or analyzed in this study. Data sharing is not applicable to this article.
